# Field evidence for transfer of plastic debris along a terrestrial food chain

**DOI:** 10.1038/s41598-017-14588-2

**Published:** 2017-10-26

**Authors:** Esperanza Huerta Lwanga, Jorge Mendoza Vega, Victor Ku Quej, Jesus de los Angeles Chi, Lucero Sanchez del Cid, Cesar Chi, Griselda Escalona Segura, Henny Gertsen, Tamás Salánki, Martine van der Ploeg, Albert A. Koelmans, Violette Geissen

**Affiliations:** 1 Agroecología, El Colegio de la Frontera Sur, Unidad Campeche, Av Polígono s/n, Cd. Industrial, Lerma, Campeche Mexico; 2 Ciencias de la Conservación, El Colegio de la Frontera Sur, Unidad Campeche, Av Polígono s/n, Cd. Industrial, Lerma, Campeche Mexico; 3 Soil Physics and Land Management Group, Wageningen University & Research, Droevendaalsesteeg 4, 6708PB Wageningen, The Netherlands; 4 Soil Quality Department, Wageningen University & Research, Droevendaalsesteeg 4, 6708PB Wageningen, The Netherlands; 5 Aquatic Ecology and Water Quality Management Group, Department of Environmental Sciences, Wageningen University & Research, P.O. Box 47, 6700 AA Wageningen, The Netherlands; 6 Wageningen Marine Research, P.O. Box 68, 1970 AB IJmuiden, The Netherlands

## Abstract

Although plastic pollution happens globally, the micro- (<5 mm) and macroplastic (5–150 mm) transfer of plastic to terrestrial species relevant to human consumption has not been examined. We provide first-time evidence for micro- and macroplastic transfer from soil to chickens in traditional Mayan home gardens in Southeast Mexico where waste mismanagement is common. We assessed micro- and macroplastic in soil, earthworm casts, chicken feces, crops and gizzards (used for human consumption). Microplastic concentrations increased from soil (0.87 ± 1.9 particles g^−1^), to earthworm casts (14.8 ± 28.8 particles g^−1^), to chicken feces (129.8 ± 82.3 particles g^−1^). Chicken gizzards contained 10.2 ± 13.8 microplastic particles, while no microplastic was found in crops. An average of 45.82 ± 42.6 macroplastic particles were found per gizzard and 11 ± 15.3 macroplastic particles per crop, with 1–10 mm particles being significantly more abundant per gizzard (31.8 ± 27.27 particles) compared to the crop (1 ± 2.2 particles). The data show that micro- and macroplastic are capable of entering terrestrial food webs.

## Introduction

Globally, hot spots of plastic pollution are confined to the oceans, landfills, open waste disposals and home gardens^1–3^. Plastic packaging constitutes 37% of all plastic wastes. Low density polyethylene is the most abundant polymer in the world^4,5^ with an estimated accumulation rate of 25 million tons per year^6^. In the Yucatan Peninsula in Southeastern Mexico, traditional Mayan home gardens in both urban and rural areas suffer from waste management problems^1^. Home gardens are agroforestry land-use systems consisting of multipurpose trees and shrubs where livestock is often raised^7^. These gardens are valued globally for their high genetic agrobiodiversity resources^8^, sustainable attributes (i.e. soil conservation^7,9^), and food security^10,11^. In developing countries such as Mexico, waste management is a big challenge due to the modification of regional consumption patterns^3^. Each person produces around 1 kg of waste daily, of which 200 g (20%) is plastic^1^. The increasing consumption of plastic bottled soft drinks not only contributes to a high risk of obesity for the local population^12^, but also to excessive plastic waste problems worldwide^13^. Home garden owners themselves are facing problems with plastic waste pollution since most of them burn their household wastes in their home gardens (leaf debris, animal wastes, plastic wastes) and bury the rest of the waste in the soil, causing soil and air pollution^1^. In aquatic ecosystems, it has been shown that micro (MP, <5 mm) and macroplastics (MaP, 5–150 mm) are taken up by different members of the food web, like lugworms, mussels and birds^14–16^. To date, studies focusing on the ingestion-egestion of plastic in terrestrial ecosystems are lacking^17,18^, which is surprising considering the potential impact of bioaccumulation of plastics on human health.

Here, we present a study that sheds light on the transfer of low-density plastic pollution through a terrestrial food web by investigating plastic pollution in Mexican home gardens. The aim of our study was to identify the transfer of low density polyethylene plastic residues in a terrestrial ecosystem, examining the egestion of plastic by the terrestrial members (earthworms and chickens (*Gallus gallus domesticus*), concentration of plastics in chicken crops and gizzards, and preparation of gizzards for human consumption.

## Results

**Figure 1:**
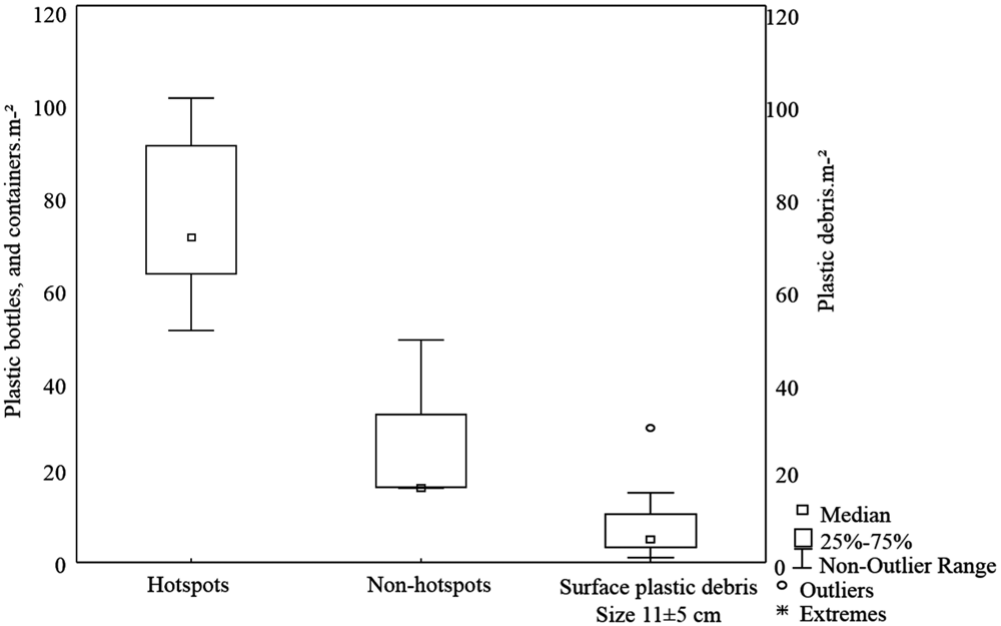
Bottles, containers and surface macroplastics per m^−2^ in home gardens. Hotspots are areas in the home gardens where plastic bottles are present in abundance.

Parts of the home gardens were covered by plastic bottles, with 74.4 ± 20.4 of PE bottles m^−2^ and 7.4 ± 6.5 particles m^−2^ of MaP found on the surface of the soil with an average size of 11 ± 5 cm per individual item (Fig. 1). MP content per soil layer and in chicken feces did not differ significantly between the home gardens.

**Figure 2:**
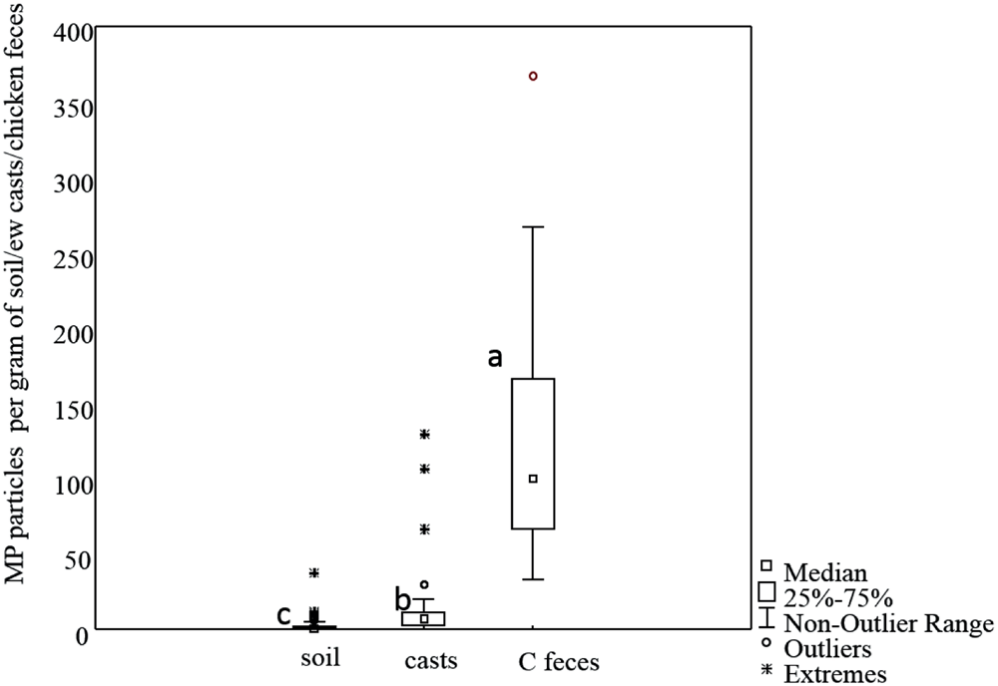
Microplastics (MPs) per gram of soil, earthworms’ casts and chicken feces. Different letters indicate significant differences among the concentration of microplastics (after Kruskal-Wallis and Mann-Whitney U test, p < 0.05).

**Figure 3:**
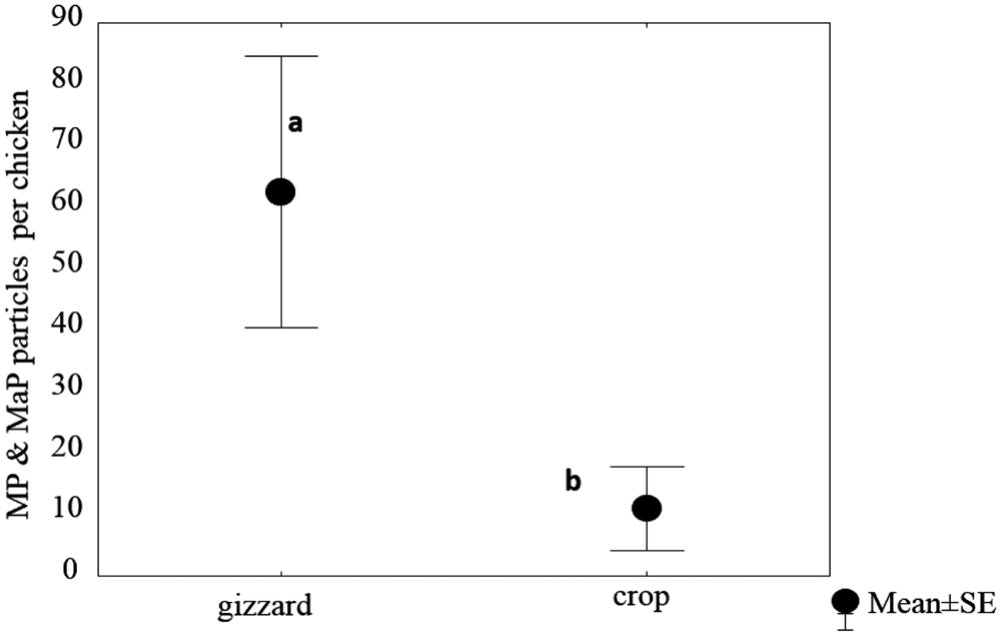
Macroplastics (MaPs) per crop and per gizzard. Different letters indicate significant differences among the concentration of macroplastics (after Kruskal-Wallis and Mann-Whitney U test, p < 0.05).

**Figure 4:**
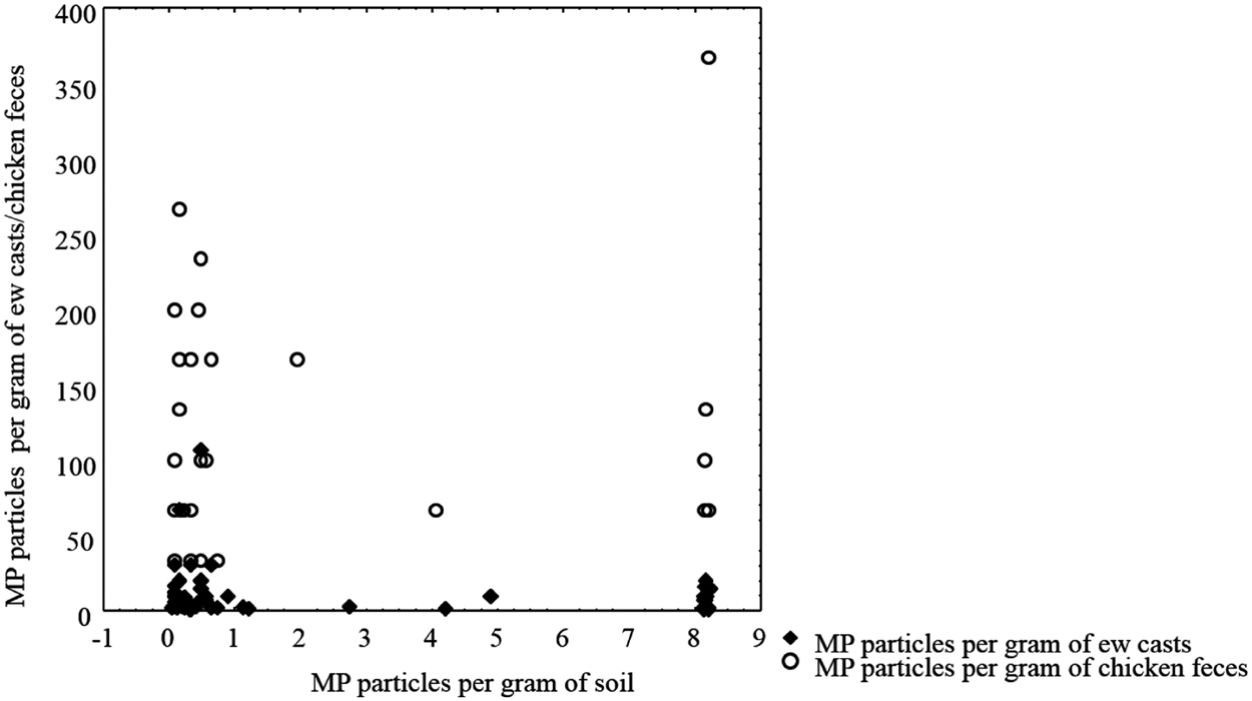
Spearman correlation between microplastics (MPs) particles per gram of soil and microplastic particles per gram of casts (r = −0.21, p < 0.05, (**a**) and between microplastics per gram of soil and microplastics per gram of feces (r = −0.01, p < 0.05 (**b**).

The number of MPs per gram of soil, casts and chicken feces was increasing in the order: soil (0.87 ± 1.9 particles g^−1^) <earthworm cast (14.8 ± 28.8 particles g^−1^) <chicken feces (129.8 ± 82.3 particles g^−1^) Figs 2 and 3. There were no significant correlations (Spearman) between MPs content in soil, casts or chicken feces (r: −0.21 and −0.01 p < 0.05 respectively, Fig. 4).

**Figure 5:**
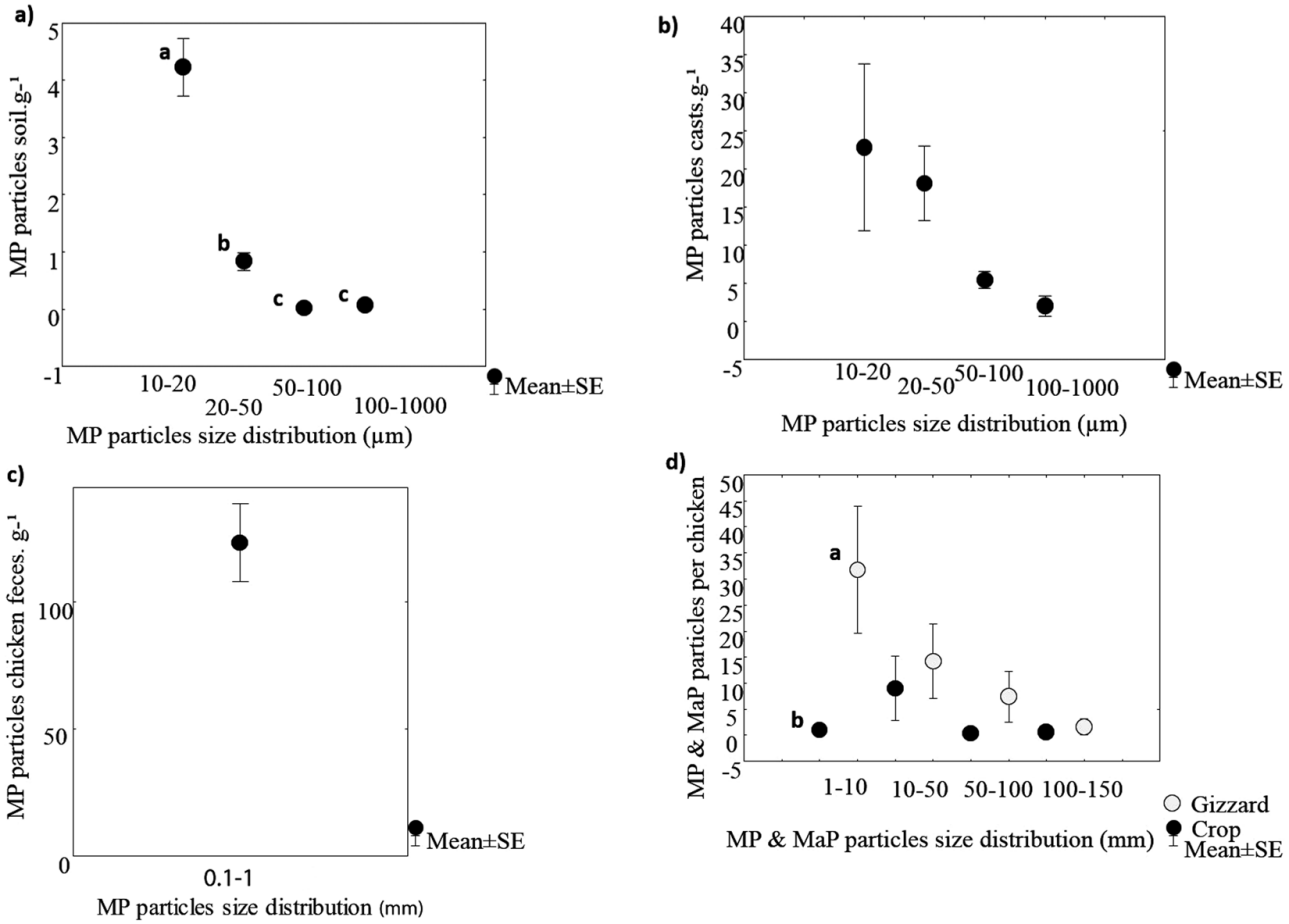
Microplastics (MPs) and Macroplastics (MaPs) size distribution per gram of soil (**a**); gram of earthworm casts (**b**); gram of chicken feces (**c**); per chicken (**d**) (crop and gizzard). Different letters indicate significant differences among the concentration of microplastics per gram at different sizes, presented in each plot (after Kruskal-Wallis and Mann-Whitney U test, p < 0.05).

The MP particle size distribution was very different in soil, cast and chicken feces. In soil, 59.3 ± 4.4% of the microplastic particles were between 10–20 μm, 34.4 ± 3.9% were between 20–50 μm, and 4.8 ± 0.2% were > 50 μm. In earthworm casts, 84.6% of the microplastic particles were between 10 to 50 μm, while 15.4% of the microplastic particles in casts were bigger than 50 μm. The percentage of microplastic particles < 50 μm in earthworm casts was similar to that of soil (80–90%). In chickens, microplastics were found only in gizzards and chicken feces. 16.45% of the plastic particles found in the gizzard were smaller than 5 mm and 83.55% were > 5 mm. In chicken crops, all plastic particles found were macroplastics and in chicken feces, all plastic particles were between 0.1 and 1 mm (microplastics, Fig. 5).

The size of the plastic debris found in chickens followed the order: crop > gizzard > chicken feces. Being the first evidence of transfer of plastic debris into chickens. The concentration ratios of MPs were 12.7 ± 9.5 for earthworm casts/soil, 105 ± 39.2 for chicken feces/soil, and 18.4 ± 22.2 for chicken feces/earthworm casts with the lowest number of MP particles found in the soil (Table S1). The gizzard/soil MP concentration ratio was 5.1 ± 6.9. MaPs were discovered in the gizzard and the crop of the chickens with a significantly higher concentration found in the gizzard (45.82 ± 42.6 MaP particles per gizzard, Chi-square 3.6 test, p: 0.05) than in the crop (11 ± 15.3 MaP particles per crop, Fig. 4). The plastic debris measuring from 1 to 10 mm found in the gizzard with a concentration of 31.8 ± 27.27 particles per gizzard was significantly higher (U test 2.19, p: 0.02) than the concentration of this particle size range found in the crop (1 ± 2.2 particles per crop, Fig. 5). The concentration ratio of MaPs in the crop and gizzards calculated with MaPs from the soil surface was 1.5 ± 2.1 in the crop and 6.1 ± 5.6 in the gizzard (Table 1).

**Table 1:** Concentration (Con) of macroplastics (MaP) from the soil surface into the crop and into the gizzard.

MaP in soil surface part.m^2^	MaP Con in crop	MaP Con in gizzard
7.4 ± 6.5	1.5 ± 2.1	6.1 ± 5.6

From the 303 plastic debris particles found in 5 chickens, 91.4% were PE-bottle debris, 6.9% fibers, and 1.7% polystyrene.

Interviews concerning the preparation of gizzards before cooking revealed that 7 out of 10 women only washed the outside of the gizzards when they prepared chicken soup and they did not clean them on the inside. The remaining 3 out of 10 women also cleaned the gizzards carefully on the inside. All the women cooked the gizzard in a soup and some of them removed the gizzard after cooking, cut it and added it to rice.

## Discussion

This is the first study that provides field evidence for the transfer of plastic debris in the terrestrial food chain, using tropical home gardens of southeast Mexico as an example. We are not aware of any similar studies for terrestrial ecosystems, thus making this the first research examining the path plastic debris takes through the food chain under realistic field conditions in tropical home gardens. We were able to carry out this research because the mismanagement of domestic waste in Mexico leads the owners of home gardens to substantially pollute their own environment.

In our study, we didn’t find significant differences among plastic particles sizes between the soil layer of 0–10 cm and the soil layer of 10–20 cm due to the fact that the soil was cultivated to a depth of 20 cm. In our study, we did not assess the presence of nanoparticles. The development of the detection of nanoplastics in soils is strongly recommended.

Chickens seem to take op plastics mainly from the plastic residues on the soil surface and therefore, MP found in soils and those found in Chicken feces were not correlated. Earthworms seem to bioconcentrate MPs stronger in casts if the MP content in soil is low. This fact was well described by Huerta *et al*. (2016). In our study, the small number of MPs found in soil (0–2 particles/g) led to a higher number of MP being detected in casts, chicken feces and chicken gizzards (ratios of 12.7, 105 and 5.1 respectively). This bioconcentration could explain the higher concentration of larger MPs being found in casts than in soil. Further studies are required in order to better understand this behavior.

Under natural conditions, earthworms ingest the equivalent of their own weight each day^19^. In a tropical home garden in Mexico, it is possible to find an earthworm biomass of 5 to 31 g m^−2,^ ^20^, which means that 5 to 31 g m^−2^ of soil is taken up daily by earthworms. The earthworm casts then concentrate the MPs present in the soil as a consequence of direct ingestion of the soil^21^ and the MPs probably accumulate in earthworm tissues^21^. Small particle selection by earthworms seem to be always present^22,23^, and reflected in our study where the highest concentration of MP per gram of cast were found within the size of 10–50 µm. Apparently, chickens mainly ingest macroplastics found on the soil surface since plastic debris >5 mm was present in the chicken crops and gizzards.

Nevertheless MPs measuring between 0.1 and 5 mm were found in the gizzard and feces. Therefore, we assume that MPs in chickens may originate from the transformation of MaPs to MPs during the passage through the digestive canal, ending up in the gizzard as a mixture of MaPs and MPs and resulting in the excrement as MPs. Under laboratory conditions, plastics ingestion by chickens reduced food consumption and the volume of the gizzards since plastic particles are well retained in the gizzards^24^. In studies of aquatic birds, some plastic-derived chemicals (ie. polybrominated diphenyl ethers) were biomagnified in their tissues (0.3–186 ng/g-lipid) while plastic debris was found in their stomachs (0.04–0.59 g/bird^25^).

The presence of MPs and/or MaPs in chicken organs (i.e. gizzards) may have negative consequences for human health^17^. Chickens are very important in the diet of the Yucatecan people^26^. This carries a potential risk to human health when local people consume polluted gizzards that are not thoroughly cleaned. Even thoroughly cleaning the gizzards would not guarantee that all of the plastic debris and chemical residues would be removed. In Mexico, chicken consumption per capita is around 15 chickens per person per year^27^. This translates into an annual possible ingestion of 840 plastic particles per person. Consumption of domestic chickens (gizzards) around the world in traditional dishes^28,29^ may potentially expose humans to high concentrations of MPs, either directly by consuming gizzards such as in this study, or indirectly through bioaugmented MPs from the chicken’s digestive system into their tissues.

## Materials and Methods

### Study site

Ten home gardens with similar vegetation and soil conditions in a rural environment with similar ethnic and economic demographics were selected as plastic hotspots in Pucnachen, Campeche, Mexico (from 20°21’50.5332”N and 90°12’57.9096”W to 20°22’00.8184”N and 90°13’ 25.7520”W). The mean annual temperature is 26 °C and the mean annual precipitation varies between 930 and 1200 mm per year^30^. The dominant vegetation is low and middle sub-caducipholy tropical rain forest^31,32^ with karstic soils^33^. These rural home gardens are common in Southeastern Mexico and are characterized by a dynamic space where there is a main house, a yard which normally contains a separate open kitchen, several plants such as shrubs and trees, as well as a livestock area and house wastes, within an average area of 50 × 50 m^1,34^.

### Sampling

Samples were taken from soil, earthworms (who ingest soil), and chickens feces and chicken crop and gizzard (who ingest earthworms and soil).

Five 50 g soil samples, taken from a depth of 0–10 and 10–20 cm, were collected from each home garden resulting in 100 soil samples in total. Earthworm casts were collected from each of the points where the soil samples were obtained. Two 10 gram samples of chicken feces were collected from each home garden. Five chickens aged between 5 and 8 months were randomly collected from the home gardens. Plastic bottles and surface MaP (>5 mm) coverage were visually assessed per m^2^.

### Plastic extraction

MPs were isolated from the soil using a new method developed for this study. Soil samples were air dried and sieved to 2 mm. Low density (<1 g.cm^3^) MPs from the soil were collected by flotation (12.3 g soil with 50 ml demineralized water) after 2 hours of ultrasonic cleaner agitation (50/60 Hz, Bath ultrasonic, Bransonic 52) and 36 hours of rest. We identified the plastic particles and distinguished them from other particles using a new technique developed and validated in our laboratory. This new technique allows us to distinguish plastic particles from soil particles by burning samples at 120 °C. Photos taken before and after burning are analyzed (Zhang *et al*. https://vimeo.com/221334286). The earthworm casts were air dried, weighed and then carefully dissolved with demineralized water (0.5 g casts with 17 ml water). The low density MPs were collected after flotation.

Chicken (*Gallus gallus domesticus*) feces were frozen in order to avoid decomposition. A homogenized sample of 0.7 g was obtained from each chicken feces sample. Demineralized water was then added (5–10 ml) to facilitate MP collection after flotation (Fig. S1).

MPs (<5 mm) and MaPs (5–150 mm) were extracted from dissected chickens using corresponding sieves according to van Franeker *et al*.^15^. The gizzard and crop were carefully washed with demineralized water. Then the contents of the gizzard and crop were left in demineralized water for 24 hours which allowed the MaPs and MPs to float to the surface of the water^32^ and then the MaPs and MPs were collected (Fig. S2), measured and counted.

### Microplastic and Macroplastic size detection and totals

MPs from soil, earthworm casts and chicken feces were counted with the use of a stereo microscope (Leica, objective10 × 21, Fig. S3) and particle size distribution was determined with a microscope (Leica, objective 40x/0.67, Fig. S4).

The floating MP (<5 mm) and MaP (5–150 mm) particles from the crop and gizzard of the chicken were placed in a glass petri dish for measurement which was carried out with the help of a millimetric paper adapted to the stereoscope. The characterization of the plastic debris was done visually.

All the plastic extractions were done in sterile conditions, using glass petri dishes, following the normal procedures of standard laboratories (using white coats and hats).

### Interviews regarding the preparation of gizzards and crops during cooking

In the Yucatan, it is mainly women who prepare the dishes that contain chicken gizzards. We interviewed ten women to assess their recipes and habits with respect to the use of gizzards and crops in cooking traditional dishes. Of special interest was the cleaning process of the gizzards in order to estimate implications for the risks of ingestion of plastic particles by humans.

## Theory and Calculations

In conducting this study, we expected to find that concentrations of low density MPs would be ingested amongst the invertebrates and vertebrates inhabiting the home gardens. We looked at soil, earthworm casts and chickens from home gardens and we expected that the lowest plastic concentrations would be found in the soil followed by higher MP concentrations in the earthworm casts and finally, in the chicken feces. We calculated three concentration ratios of MPs which refer to the concentrations of MPs between earthworm casts and soil, between chicken feces and soil and between chicken feces and earthworm casts. Furthermore, we estimated the concentration ratio between MPs and MaPs in chicken gizzards and crops and MPs in the soil layers and the surface soil MaPs. Due to the lack of statistically significant differences in soil plastic particle concentration and plastic size distribution between the two studied layers, the calculations were done with the information from both soil layers.

### Microplastic (MP) concentration ratios soil/ chicken feces

In order to calculate the number concentration ratio of MPs per g casts/MPs per g soil, a number concentration ratios were determined using the followed equation:

1 $${\rm{Ccs}}=\frac{CMPc}{CMPs}$$

where Ccs is the number concentration ratio of MP particles per g of earthworm casts/ particles per g soil, CMPc is the MP particle number per g cast, and CMPs is the MP particle number per g soil. In order to calculate the number concentration ratio of MPs between chicken feces and soil, we used the following equation:

2 $$Cchs=\frac{Cpch}{CMPs}$$

where Cchs is the concentration ratio of MP particles per g chicken feces/ particles per g soil, and Cpch is the particle number g^−1^of MPs in chicken feces.

To determine the number concentration ratio of MPs from chicken feces/ earthworm casts (Cchc), we used the following equation:

3 $$Cchc=\frac{Cpch}{CMpc}$$

### Microplastic concentration ratio soil/ chicken gizzard

In order to obtain the concentration ratio of MPs found in the chicken gizzard (CMPg) the followed equation was used:

4 $$CMPg=\frac{MPg}{{\rm{CMPs}}}$$

where MPg is the MP particle number per gizzard unit, and CMPs is the number of MP particles per g soil.

### Macroplastic concentration ratio soil surface/ chicken gizzard and crop

In order to determine the concentration ratio of MaPs per gizzard unit/ MaPs per m^2^ soil surface the following equation was used:

5 $$CMaPg=\frac{MaPg}{{\rm{MaPsf}}}$$

where CMaPg is the concentration ratio of MaPs per gizzard unit (MaPg, average number of particles /unit of the gizzard) and MaPs per m^2^ soil surface (MaPsf, average particle number m^−1^ soil surface). The concentration ratio of MaPs between crops and soil surface (CMaPc), was calculated as:

6 $$CMaPc=\frac{MaPc}{{\rm{MaPsf}}}$$

where MaPc is the average of the concentration of MaPs found in the crop.

### Statistics

The collected data did not follow a normal distribution pattern (KS test). Therefore, non-parametric Kruskal – Wallis H-test for multiple comparisons followed by the Mann Whitney U test for pairwise comparisons were performed in order to determine the significance of the differences among MP content in soil, cast and chicken feces. A Kruskal-Wallis H-test was performed to determine significant differences in the MP content in the different soil layers as well as in the chicken feces among the home gardens. A Mann Whitney U test was done to compare the amount of plastic debris between crops and gizzards, and between the plastic debris sizes in soils, casts, and chickens. We also conducted Spearman correlation analysis for MP content in soil-cast, and MP content in soil and chicken feces. We used Statistica Software (version 2015).

### No ethical approval and informed consent was required

Due to the fact that this study was developed only with dead chickens, no need for an ethical approval was required. In this study dead chickens were opened according to van Franeker *et al*.^15^.

### Data availability

The datasets generated during and/or analysed during the current study are available from the corresponding author on reasonable request.

Data will be available (if required) through DANS EASY (https://easy.dans.knaw.nl/ui/home) or a data repository preferred by Scientific reports.

## Electronic supplementary material

Text S1
