# Supplementary material for: Field evidence for transfer of plastic debris along a terrestrial food chain
Source: Sci Rep. 2017 Oct 26;7:14071. doi: 10.1038/s41598-017-14588-2 (PMC5658418; doi:10.1038/s41598-017-14588-2)
Supplement: Supplementary file 1 — Text S1 [file 41598_2017_14588_MOESM1_ESM.pdf]

**Supplementary Materials**

Tables S1-S#

Table S1. Concentration ratios of Microplastic (MP) particles between the chicken feces and the soil, the chicken feces and the earthworms casts, and the earthworms cast and the soil. Ccs: concentration ratio between the concentration of MPs in casts and the concentration of MPs in casts, Cchs concentration of MPs between the concentration of MPs in chickens feces and the concentration of MPs in earthworms casts. Cchc: concentration ratio between the concentration of MPs in chicken feces and the concentration of MPs in earthworm casts. CMPg: concentration ratio of MPs in chicken gizzard and MPs in soil.

| $MP.g^{-1}$ soil | Ccs | Cchs | Cchc | CMPg |
|---|---|---|---|---|
| 0-2 | 12.7±9.5 | 105±39.2 | 18.4±22.2 | 5.1±6.9 |
| 2-3 | 3.2±1 | 30.3±10.4 | 9.5±0 | 3.4±4.6 |
| 3-6 | 3.1±1.9 | 30.4±9.8 | 13.9±8 | 2.2±3.1 |

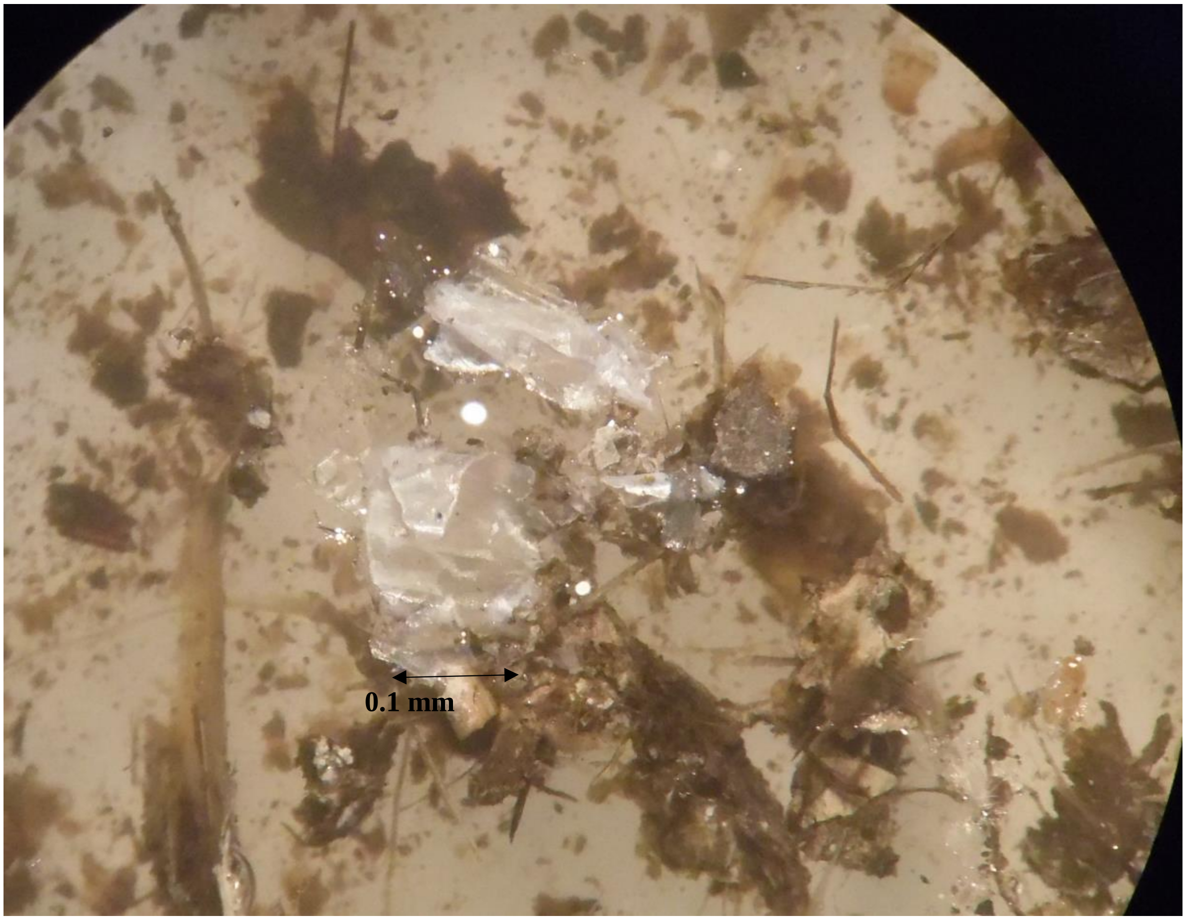


Fig. S1. Microplastic particles photograph obtained from chicken feces from Home gardens.

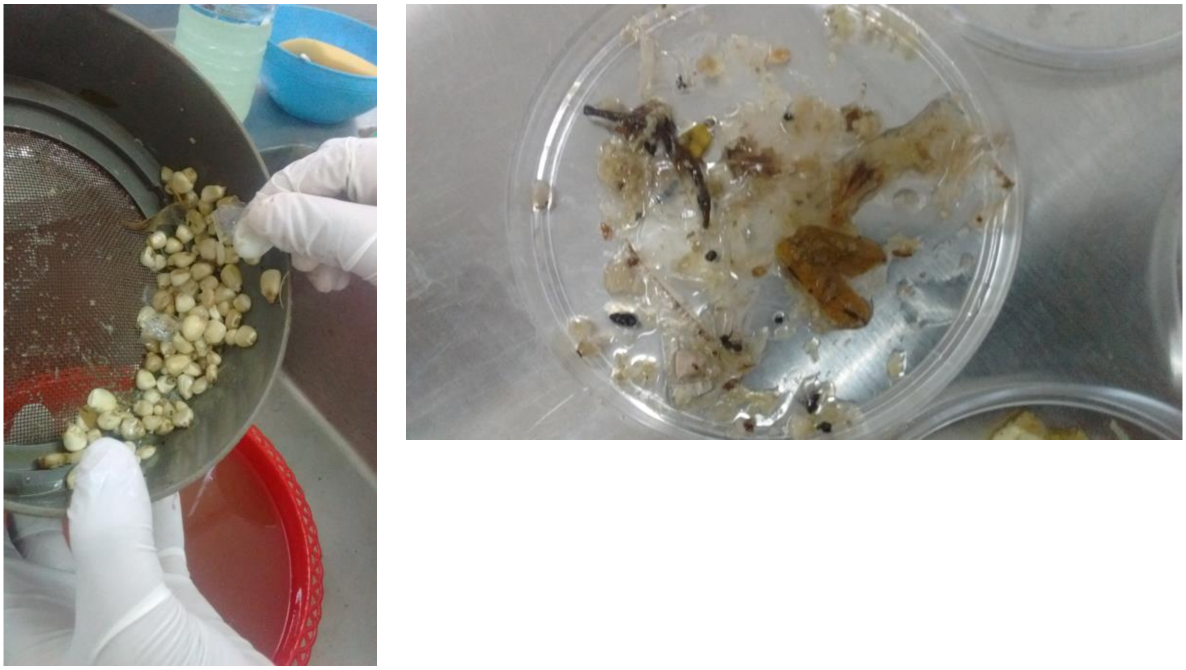

Fig. S2. Macroplastic particles collected from chicken crop and gizzard.

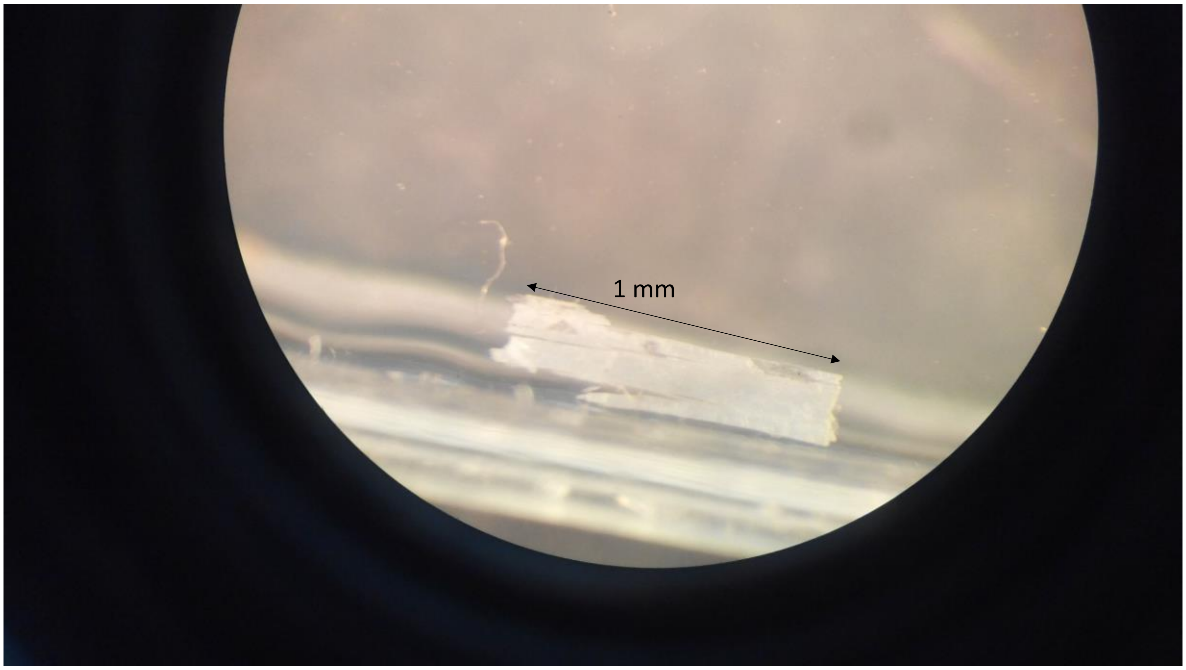


Fig. S3. Microplastic particles photograph obtained from soils of the Home gardens. Stereo microscope Leica, objetive10x21.

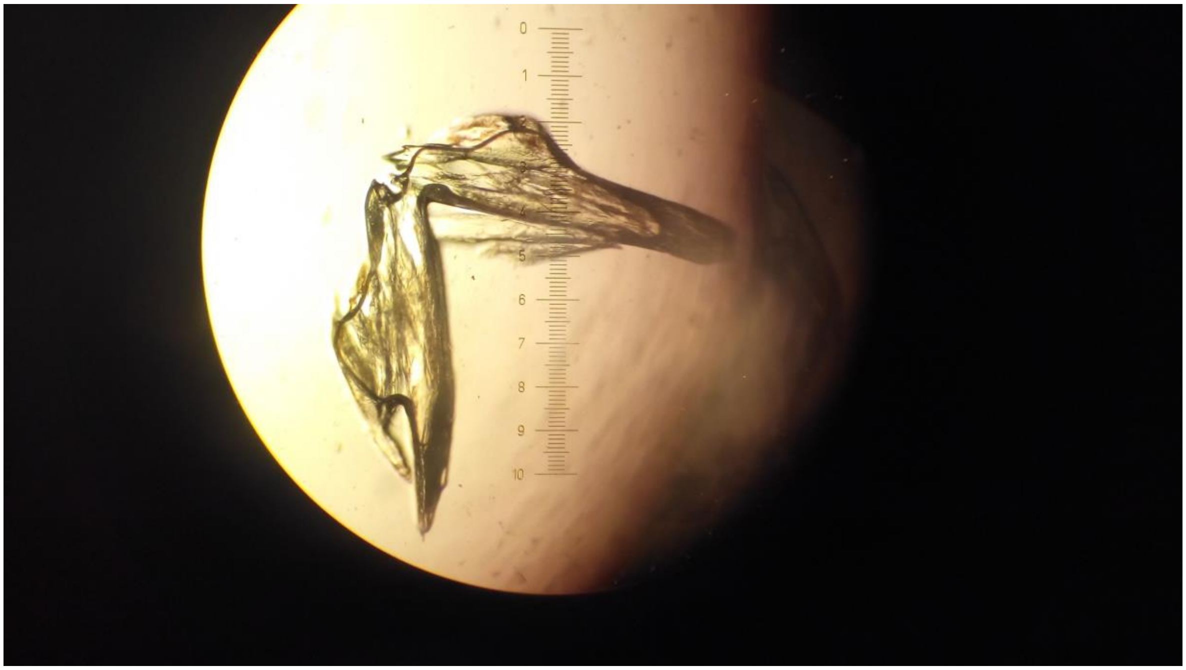


Fig. S4. Microplastic particle photographs obtained from soils of the Home gardens. Microscope Leica, objective 40x/0.67 (each line from the ruler = 10 µm).
